# Autophagy Regulates Putative Anion Transporter 1 Expression in Intestinal Epithelial Cells

**DOI:** 10.1111/jcmm.70513

**Published:** 2025-05-02

**Authors:** Shubha Priyamvada, Dulari Jayawardena, Arivarasu N. Anbazhagan, Anoop Kumar, Seema Saksena, Ravinder K. Gill, Alip Borthakur, Waddah A. Alrefai, Pradeep K. Dudeja

**Affiliations:** ^1^ Division of Gastroenterology & Hepatology, Department of Medicine University of Illinois Chicago Chicago Illinois USA; ^2^ Jesse Brown VA Medical Center Chicago Illinois USA; ^3^ Department of Clinical and Translational Sciences Marshall University Huntington West Virginia USA

**Keywords:** autophagy, intestine, post‐translational regulation, putative anion transporter 1

## Abstract

Putative anion transporter 1 (PAT‐1) is the key oxalate‐secreting transporter in the intestine and therefore, maintaining its steady‐state levels is critical for oxalate homeostasis. Autophagy is known to modulate the expression of intestinal solute transporters; however, its role in regulating PAT‐1 has not been examined. Autophagy in Caco‐2 cells was induced by either rapamycin treatment or by nutrient deprivation and assessed by conventional autophagy marker proteins. ATG7 (autophagy‐related 7) protein expression was attenuated by ATG7‐siRNA in Caco‐2 cells or by utilising ATG7KO mice. PAT‐1 protein levels in Caco‐2 cells were significantly reduced by rapamycin or by nutrient deprivation at 48 and 72 h. Concomitantly, the LC3II/I ratio was increased, and p62 levels were significantly decreased, confirming the induction of autophagy. Nutrient deprivation for 6 h also caused a significant decrease in the surface levels of PAT‐1. PAT‐1 protein levels were increased by the siRNA‐mediated ATG7 knockdown in Caco‐2 cells and in the ileum of ATG7KO mice. In summary, Autophagy in intestinal epithelial cells modulates the basal levels of PAT‐1 protein and may play a critical role in the maintenance of oxalate homeostasis.

**Abbreviations**
ATG7: Autophagy‐related gene 7
ATP: adenosine triphosphate
BSA: bovine serum albumin
CFTR: cystic fibrosis transmembrane conductance regulator
EBSS: Earle's Balanced Salt Solution, nutrient deprivation medium
ECL: enhanced chemiluminescence
GAPDH: glyceraldehyde‐3‐phosphate dehydrogenase
GLUT1: glucose transporter 1
Hs: human (Homo sapiens)
IBD: inflammatory bowel disease
IECS: intestinal epithelial cells
IFN‐γ: interferon‐γ
KO: knockout
LC3: microtubule‐associated protein light chain 3
m: mouse
mTOR: mammalian target of rapamycin
NGS: normal goat serum
NHE3: Na^+^/H^+^ exchanger 3
PAT‐1: putative anion transporter‐1
PBS: phosphate buffered saline
PE: phosphatidylethanolamine
SDS‐PAGE: sodium dodecyl‐sulfate polyacrylamide gel electrophoresis
SLC26: solute carrier family 26
SLC2A1: solute carrier family 2, member 1
SLC9A3: solute carrier family 9 member A3
SQSTM1: sequestosome 1
TBST: Tris buffered saline with Tween 20
TNF‐α: tumour necrosis factor‐α
Ubc: Ubiquitin C
WT: wild type

## 1 Introduction

Putative anion transporter 1 (PAT‐1 or SLC26A6) is an apical membrane anion exchanger with a higher level of expression in the mouse small intestine compared to the colon [1] where it is abundantly expressed on the apical membrane of intestinal villi, extending from the duodenum to the jejunum and ileum. Primarily identified to function as a Cl^−^/HCO_3_^−^ exchanger in facilitating electroneutral NaCl absorption, PAT‐1 is now believed to be a major oxalate‐secreting transporter in the small intestine via its chloride/oxalate exchange activity [2, 3, 4].

*Slc26a6*‐null mice exhibit defects in intestinal oxalate secretion, resulting in enhanced net absorption of ingested oxalate, hyperoxalemia, hyperoxaluria and a high incidence of kidney stones [5, 6]. Further, mutations in the SLC26A6 gene have been shown in patients with kidney stones [7, 8, 9, 10]. Notably, a recent study identified a dominant negative mutation in the human SLC26A6 gene that impairs the transport function and membrane expression of SLC26A6, representing a significant risk factor for inherited forms of hyperoxaluria and nephrolithiasis [10]. Studies have also shown that reduced PAT‐1 function and expression in the intestine contribute to obesity‐associated hyperoxaluria in obese rodent models [11, 12]. These studies demonstrate that PAT‐1 plays a crucial role in oxalate homeostasis and has emerged as a novel therapeutic target for calcium‐oxalate nephrolithiasis, a kidney disease linked to defective oxalate secretion [13, 14].

In view of the emerging significance of intestinal PAT‐1 in normal physiology and pathophysiology of kidney diseases, an in‐depth understanding of the mechanisms of its regulation under normal and disease conditions is warranted. In this regard, in vitro studies in the intestinal epithelial cells have shown the downregulation of PAT‐1 in response to nucleotide (ATP and UTP) treatment [15] and stimulation of cholinergic signalling [16]. Studies by our group further showed a transcriptional and post‐transcriptional downregulation of PAT‐1 expression by proinflammatory cytokine interferon‐γ [17] and microRNA125a‐5p [18], respectively. Also, an in vivo study showed an increase in apical membrane expression of PAT‐1 by Lubiprostone [19]. In addition, *Oxalobacter formigenes* ‐derived bioactive factors prevent urinary oxalate excretion in hyperoxaluric mice [20] via enhancing PAT‐1 function. Also, short‐chain fatty acids [21] were shown to reduce renal calcium oxalate stone formation in rats via upregulation of PAT1. These reports demonstrated that PAT‐1 function/protein expression can be modulated via both transcriptional and post‐translational mechanisms. However, there are very limited studies available focusing on the regulation of PAT‐1 function and expression under basal conditions.

In this regard, autophagy, an intracellular renewal process for degradation and recycling of proteins and organelles [22, 23], is pivotal in maintaining cellular homeostasis. In the intestinal epithelium, autophagy has been shown to play a critical role in regulating the anti‐microbial defence, gut‐microbe interactions, mucosal immune response and epithelial barrier integrity [24, 25]. Recent studies showed autophagy to modulate intestinal epithelial barrier function via its effects on tight junction proteins [26, 27] and to alter the expression/activity of intestinal ion/solute transport proteins such as SLC9A3 (NHE3) [28], SLC2A1 (GLUT1) [29] and the chloride channel CFTR [30]. For example, NHE3 expression is significantly downregulated in response to rapamycin, a potent inducer of autophagy [28]. Also, the deletion of ATG7 (an important autophagy protein) in mice led to an increase in total NHE3 levels in this study, suggesting that basal autophagy plays a role in regulating NHE3 expression [28]. Similarly, inhibiting mTOR (mammalian target of rapamycin), a key regulator of autophagy, has been shown to enhance the stability and function of CFTR by promoting membrane trafficking through the stimulation of autophagy [30]. Additionally, under metabolic stress states that demand a higher availability of glucose, an increase in GLUT1 expression at the cell surface, along with enhanced glucose uptake, has been demonstrated to be mediated by autophagy [29]. Collectively, these studies highlight the importance of autophagy in regulating intestinal ion transport.

The present study investigated the role of autophagy in the potential regulation of PAT‐1 expression under basal conditions. We observed that augmentation of autophagy in vitro in intestinal epithelial cells resulted in a significant decrease in total PAT‐1 protein levels. Our results also showed that inhibition of autophagy: (a) in vitro by siRNA‐mediated knockdown of ATG7; or (b) in vivo in Atg7 deficient mice resulted in upregulation of PAT‐1 expression. These findings demonstrate the role of autophagy in the post‐translational regulation of PAT‐1 expression in intestinal epithelial cells.

## 2 Materials and Methods

### 2.1 Reagents and Antibodies

Eagle's minimum essential medium (EMEM) and Caco‐2 cells were obtained from ATCC (American Type Culture Collection, Manassas, VA). Earle's balanced salt solution (EBSS, Sigma, catalogue no. E3024) was used to induce starvation. The autophagy inducer, rapamycin, was procured from Invitrogen (catalogue no. PHZ1235). Primary antibodies for LC3II/I (cat. no. 12741) p62 (cat. no. 7695), and ATG7 (cat. no. 8558) were procured from Cell Signalling Technology (Beverly, MA). The PAT‐1 antibody was a generous gift from Dr. Peter S. Aronson (Yale University, New Haven, CT), which is an affinity‐purified rabbit polyclonal antibody (R29; Pocono Rabbit Farm and Laboratory, Canadensis, PA) directed against a 22 amino acid peptide (CDLRRRDYHMERPLLNQEHLEE) from the NH2‐terminal region of human SLC26A6 [31, 32]. The secondary antibodies HRP‐conjugated goat anti‐mouse (cat. no. W4021) and HRP‐conjugated goat anti‐rabbit IgG (H + L) (cat. no. W4011) were purchased from Promega (Madison, WI). All other chemicals were of at least reagent grade and were obtained from either Sigma Chemicals (St. Louis, MO) or Fisher Scientific (Pittsburgh, PA).

### 2.2 Cell Culture

For the in vitro studies, Caco‐2 cells cultured in T‐150 (150 cm^2^) plastic flasks at 37°C in a 5% CO2, 95% O2 environment were used. The cells are cultured in EMEM supplemented with 10% foetal bovine serum, 100 IU/mL penicillin and 100 μg/mL streptomycin. Cells used for these studies were between passages 25 and 45 and were plated on 24‐well plates on plastic supports at a density of 2 × 10^4^ cells/well. Experiments involving Caco‐2 cells were done 12–14 days post‐plating to allow differentiation before treatments. For induction of autophagy, differentiated Caco‐2 monolayers were incubated with EBSS or rapamycin (500 nM) for 24, 48, and 72 h for long‐term studies. For cell surface biotinylation, cells were given EBSS treatment for 3 h or 6 h as indicated in the results section.

### 2.3 In Vivo Studies

To examine the role of autophagy in modulating PAT‐1 in the native intestine, mice with inducible knockdown of the key autophagy gene Atg7 were used. The mouse model was generated by crossing Atg7^fl/fl^ mice with mice carrying a Ubc‐CreERT2 allele [tamoxifen‐regulated Cre recombinase fusion protein under the control of the ubiquitously expressed ubiquitin C (Ubc) promoter]. Global deletion of ATG7 in adult mice (10 weeks old) was achieved by tamoxifen administration. Ileal tissue (used for RNA and protein extraction) and OCT sections of age‐matched ATG7 floxed [Atg7^fl/fl^ referred to as WT (wild type)] and Atg7^−/−^ [referred to as ATG7KO (ATG7 knockout)] were a generous gift from Dr. Nighot from the Pennsylvania State College of Medicine, Hershey, PA, USA [33]. Real‐time qPCR, western blotting, and immunofluorescence were performed as described below.

### 2.4 Preparation of Cell/Tissue Lysate (Caco‐2 and Ileal Tissue)

Lysates of Caco‐2 cells and ileal tissue were made as previously described by us [34, 35, 36]. Briefly, post‐treatment/siRNA transfection, Caco‐2 cells were washed with ice‐cold 1XPBS twice and solubilised in cell lysis buffer (Cell Signaling, Danvers, MA) supplemented with protease inhibitor mixture (Roche Applied Science, Madison, WI) and phosphatase inhibitor cocktail 3 (Sigma Aldrich). Cells suspended in lysis buffer were sonicated (three pulses for 20 s each) followed by centrifugation at 13,000 rpm for 7 min at 4°C to remove cell debris. The clear supernatant was saved for western blotting. For tissue lysates, ileal tissues were suspended in RIPA lysis buffer (Cell Signaling, Danvers, MA). Protein concentrations in the lysates were measured by the Bradford reagent obtained from Bio‐Rad (Hercules, CA) [37].

### 2.5 Western Blotting

For western blotting, 20–50 μg of cell or tissue lysates were boiled in SDS‐gel loading buffer for 5 min and subjected to electrophoresis in a 4%–15% gradient SDS‐polyacrylamide gel (Bio‐Rad) at 100 V until fully resolved. The separated proteins were transferred onto a PVDF membrane (Bio‐Rad). Membranes were blocked in the blocking buffer [5% nonfat dry milk in 1XPBS or 3% BSA (Sigma) in 1XTBS (Tris Buffered Saline)] at room temperature, followed by primary antibody incubation overnight at 4°C with anti‐PAT‐1 (1:13,000; 1%‐PBS milk); LC3 (1:500, 1%BSA‐1XTBS‐0.1% Tween‐20); p62 (1:500, 1%BSA‐1XTBS‐0.1% Tween‐20); GAPDH (1:10,000; 1%‐PBS milk). After washing in wash buffer (1X‐TBS or 1xPBS‐0.1% Tween 20) 5 times for 5 min each, membranes were then probed with horseradish peroxidase‐conjugated goat anti‐rabbit or anti‐mouse IgG secondary antibody (1:10,000) diluted in 1% BSA‐TBST or 1%‐1X‐PBS milk for 1 h. All incubations and washes were performed with gentle agitation on a rocker. Bands were visualised using Enhanced Chemiluminescence detection reagents (ECL, Bio‐Rad) using the ChemiDoc XRS+ imager and Image Laboratory 5.0 software (Bio‐Rad). Band intensities were measured using Image J software (NIH, Bethesda, MD, USA) for densitometric analysis.

### 2.6 RNA Isolation and mRNA Expression

RNA was isolated from Caco‐2 cells and mouse intestinal tissues using the RNeasy Mini Kit (Qiagen) according to the manufacturer's instructions. An equal amount of RNA (75 ng of RNA in a 10‐μL total volume) for each sample was reverse‐transcribed and amplified in a one‐step reaction using the Brilliant III Ultra‐Fast SYBR Green master mix (Agilent). The thermocycling and amplification measurements were made using the Mx3000p qPCR system (Stratagene, San Diego, CA). Changes in mRNA levels were quantified using the ΔΔCt method with GAPDH (glyceraldehyde‐3‐phosphate dehydrogenase) mRNA as an internal control.

The gene‐specific primers for human (Hs) or mouse (m) ATG7, GAPDH, and human PAT‐1 used for the RT‐PCR reactions are as in Table 1.

**TABLE 1:** Primers used in the study.

Gene	Primer sequence
Human	
HsATG7‐Forward	5′‐ATGATCCCTGTAACTTAGCCCA‐3′
HsATG7‐Reverse	5′‐CACGGAAGCAAACAACTTCAAC‐3′
HsPAT‐1‐Forward	5′‐AGATGCCCCACTACTCTGTCCT‐3′
HsPAT‐1‐Reverse	5′‐ATCCACACCACACCTCTGCTT‐3′
HsGAPDH‐Forward	5′‐GAAATCCCATCACCATCTTCC‐3′
HsGAPDH‐Reverse	5′‐AAATGAGCCCCAGCCTTCT‐3′
Mouse	
mGAPDH‐Forward	5′‐AGGTCGGTGTGAACGGATTTG‐3′
mGAPDH‐Reverse	5′‐TGTAGACCATGTAGTTGAGGTCA‐3′
mATG7‐Forward	5′‐GTTCGCCCCCTTTAATAGTGC‐3′
mATG7‐Reverse	5′‐TGAACTCCAACGTCAAGCGG‐ 3′

### 2.7 Cell Surface Biotinylation

Biotinylation studies were performed to assess autophagy‐induced modulations in the surface levels of PAT‐1 as previously described by us [38, 39]. Post EBSS treatment, Caco‐2 monolayers were incubated with sulpho‐NH‐SS‐biotin (1.5 mg/mL; Thermo Fisher Scientific) in borate buffer (in mM: 154 NaCl, 7.2 KCl, 1.8 CaCl_2_, and 10 H_3_BO_3_, pH 9.0) for 60 min to label the cell surface antigens. The procedure of labelling was performed at 4°C to prevent cellular recycling. Equal amounts of protein lysates (biotinylated) were mixed with neutravidin plus ultra‐link resin (Cat. No. 53151, Thermo Fisher Scientific) to facilitate the pull‐down of biotinylated antigens. Biotinylated proteins were released by boiling in Laemmli buffer containing dithiothreitol. Released proteins were subjected to SDS‐PAGE and transferred to PVDF membranes. Western blotting was used to compare surface PAT‐1 with intracellular fractions (not removed by avidin precipitation method) and total cellular PAT‐1 levels.

### 2.8 siRNA Silencing of ATG7

To inhibit autophagy, the expression of ATG7 (essential for autophagosome formation) was attenuated using ATG7‐specific siRNA [100 pmole, the target sequence of Hs_ATG7L_5 FlexiTube siRNA (Cat no. SI02655373): ATCAGTGGATCTAAATCTCAA] from Qiagen (Valencia, CA). Scrambled (control) siRNA (100 pmole) was used as a negative control. Caco‐2 cells were plated on 6‐well plates at a density of 1 × 10^5^ cells/well, 24 h before transfection. Cells were transiently transfected with scrambled and ATG7 siRNA using Lipofectamine 2000 transfection reagent (Invitrogen) as recommended by the manufacturer. Transfected cells were harvested 72 h post‐transfection and lysed to isolate the proteins and RNA as described above. ATG7 silencing was validated by real‐time PCR and western blotting.

### 2.9 Immunofluorescence Staining

Cryosections (5 μm) from the ileum of WT and ATG7 KO mice were stained as described previously [39] with some modifications. Briefly, tissue sections were fixed with 4% PFA followed by permeabilization with NP‐40 (0.03%, Sigma) and blocking with 5% NGS for 2 h at room temperature. The sections were stained with the following primary antibodies (1:100 in 1% NGS in 1X PBS): PAT‐1 [affinity‐purified rabbit polyclonal antibody, custom synthesised against 22 amino acid peptide (CDLRRRDYHMERPLLNQEHLEE) from the NH2‐terminal region of human SLC26A6, Pocono Rabbit Farm and Laboratory, Canadensis, PA Pocono rabbit farms] or villin (Invitrogen) and secondary antibodies: alexa Fluor 488‐conjugated goat anti‐rabbit IgG (green) and Alexa Fluor 568‐conjugated goat anti‐mouse IgG (red). The slides were mounted with SlowFade Diamond antifade reagent using a coverslip, and images were acquired using a fluorescence microscope (Zeiss). PAT‐1 fluorescence intensity in WT and ATG7KO mice was measured using the ImageJ software (NIH, Bethesda, MD, USA).

### 2.10 Statistical Analysis

Results are expressed as mean ± SEM. Each independent set represents the mean ± SEM of data from at least 6 wells used on 3 separate occasions. Differences between control versus various treatments were analysed using (a) One‐way analysis of variance (ANOVA) with Tukey test to analyse the differences between more than two groups or (b) Two‐tailed Student's *t* test for two groups. Data sets were analysed using Prism (GraphPad Software version 6). Differences were considered significant at *p* < 0.05.

## 3 Results

### 3.1 Nutrient Deprivation Decreases PAT‐1 Protein Expression in Caco‐2 Cells

**FIGURE 1:**
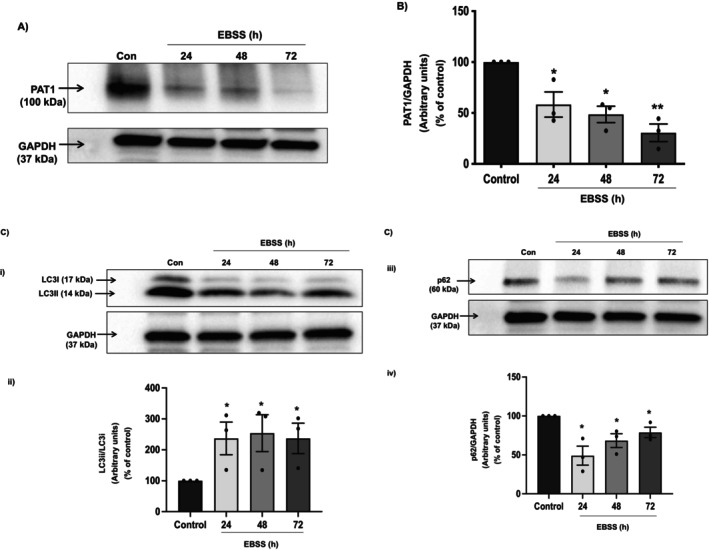
Nutrient starvation‐induced autophagy promotes time‐dependent degradation of PAT‐1 protein. Caco‐2 cells were incubated in nutrient deprived medium and expression of PAT‐1 and autophagy markers LC3 (I and II) and p62 were analysed at indicated time points. Band intensities for PAT‐1 (A), LC3 (I and II) (Ci) and p62 (Ciii); densitometric analyses to determine the change in relative levels of PAT‐1 (B), ratio of LC3II/LC3I (Cii) and p62 (Civ); GAPDH was used as internal control. *n* = 3, representative blots and images are shown. Results are mean ± SEM analysed by one‐way ANOVA. *p < 0.05 and **p < 0.01 compared to control.

Nutrient deprivation is a potent model for induction of autophagy [26, 40]. Therefore, to examine the effect of autophagy on PAT‐1 expression, a time course experiment was performed where differentiated Caco‐2 cells were incubated with EBSS (nutrient‐deprived medium) for 24, 48, and 72 h. As shown in Figure 1A, induction of autophagy resulted in a marked decrease in PAT‐1 protein levels at 24 h. Densitometric analysis revealed ~50% (*p* < 0.05, Figure 1B) decrease in PAT‐1 protein level compared to the control. This reduction in PAT‐1 protein levels was sustained for 72 h. LC3, the mammalian homologue of the yeast autophagy protein ATG8, is highly upregulated under starvation conditions [41]. Upon autophagy induction, LC3‐I is converted to its lipidated form (LC3‐II) by conjugation with phosphatidylethanolamine (PE). PE facilitates LC3‐II binding with the autophagosomal membrane [26, 41]. The conversion of soluble LC3‐I to lipid‐bound LC3‐II is considered a marker for autophagy induction. To validate the induction of autophagy, we measured the ratio of LC3II/LC3I in EBSS‐treated Caco‐2 cells. Starvation resulted in an increase in LC3II/LC3I ratio (*p* < 0.05), indicative of autophagy induction (Figure 1Ci,ii). Additionally, we also noted a reduction in levels of p62 [also called sequestosome 1 (SQSTM1)] in response to EBSS treatment (Figure 1Ciii,iv). p62 promotes linking ubiquitinated proteins to the autophagic machinery, enabling lysosomal degradation. Reduction in p62 levels marks an increase in the autophagic process.

### 3.2 Autophagy Induction by Rapamycin Negatively Regulates PAT‐1 Protein Expression

**FIGURE 2:**
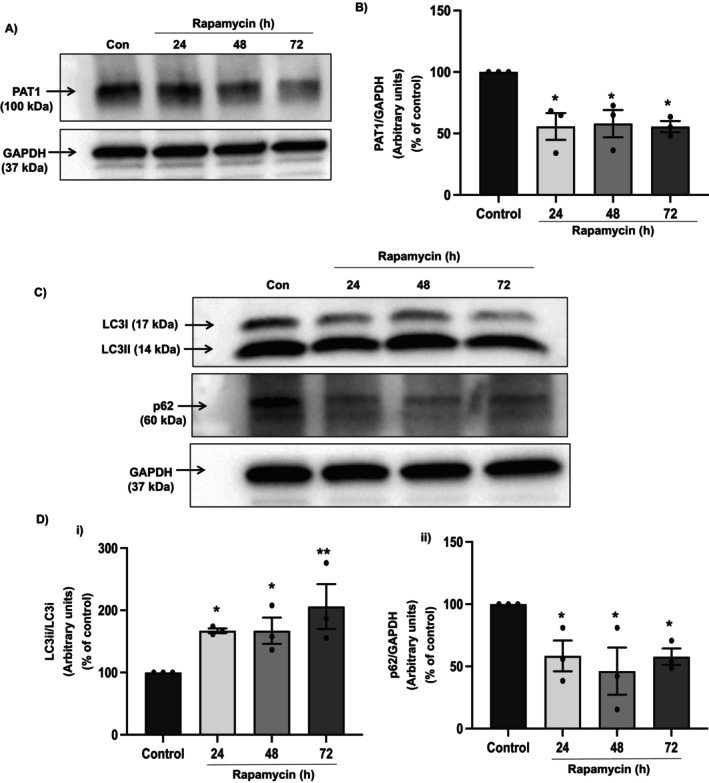
Autophagy inducer rapamycin negatively regulates PAT‐1 protein expression. Caco‐2 cells were incubated in presence of rapamycin and expression of PAT‐1, LC3 (I and II) and p62 was analysed at indicated time points. Band intensities for PAT‐1 (A), LC3 (I and II) and p62 (C); Densitometric analyses to determine change in relative levels of PAT‐1 (B), ratio of LC3II/LC3I, marker for autophagy induction (Di) and p62 (Dii). GAPDH was used as internal control. *n* = 3, representative blots and images are shown. Results are mean ± SEM analysed by one‐way ANOVA. *p < 0.05 and **p < 0.01 compared to control.

The mammalian Target of Rapamycin (mTOR) signalling pathway plays a key role in transmitting autophagic stimuli because of its ability to sense metabolic and hormonal signals. Inactivation of mTOR by treatment with the drug rapamycin induces autophagy despite ample nutrients [28, 42, 43]. To induce autophagy by pharmacologic means, differentiated Caco‐2 cells were exposed to rapamycin for 24 h, 48 h, and 72 h. Interestingly, in line with the results obtained with the nutrient deprivation‐induced autophagy model, PAT‐1 protein levels significantly decreased in response to rapamycin treatment (Figure 2A). Densitometric analysis of the protein band showed that Rapamycin decreased PAT‐1 protein levels (~50%) compared to control (Figure 2B). Pronounced increase (*p* < 0.05 & *p* < 0.01) in the ratio of lipidated LC3II/LC3I (Figure 2C,Di) and a decrease in p62 protein levels (Figure 2C,Dii) confirmed the induction of autophagy by rapamycin at all the treatment time points.

**FIGURE 3:**
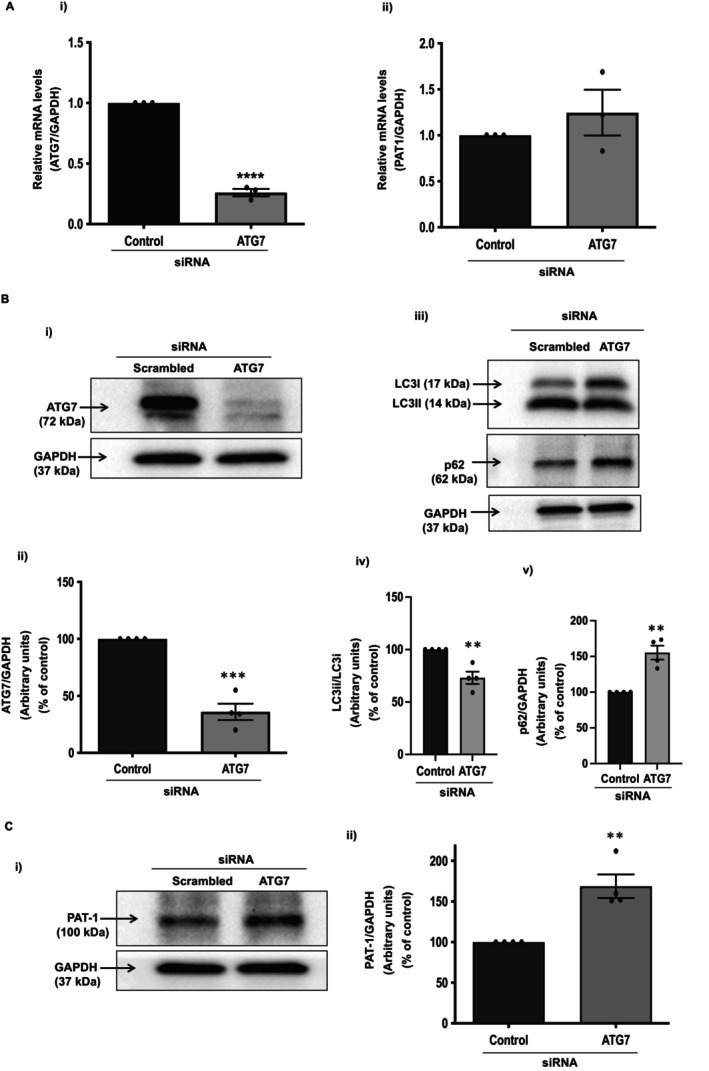
Inhibition of autophagy enhances PAT‐1 protein expression. Autophagy in Caco‐2 cells was inhibited by siRNA silencing of autophagy‐related protein ATG7. Knockdown of ATG7 at mRNA (Ai) and protein level (band intensities, Bi; densitometric analysis, Bii) was determined by qPCR and western blot, respectively. ATG7 silencing inhibited autophagy by restricting the conversion of LC3i to LC3ii (band intensities, Biii; densitometric analysis, Biv) and leading to the accumulation of p62 protein (western blot, Biii; densitometric analysis, Bv). ATG7 siRNA treatment caused a marked increase in baseline PAT‐1 protein levels (band intensities, Ci; densitometric analysis, Cii) with no effect on PAT‐1 mRNA expression (Aii). GAPDH was used as internal control. *n* = 3–4, representative blots and images are shown. Results are mean ± SEM analysed by one‐way ANOVA. **p < 0.01, ***p < 0.001 and ****p < 0.0001 compared to control.

**FIGURE 4:**
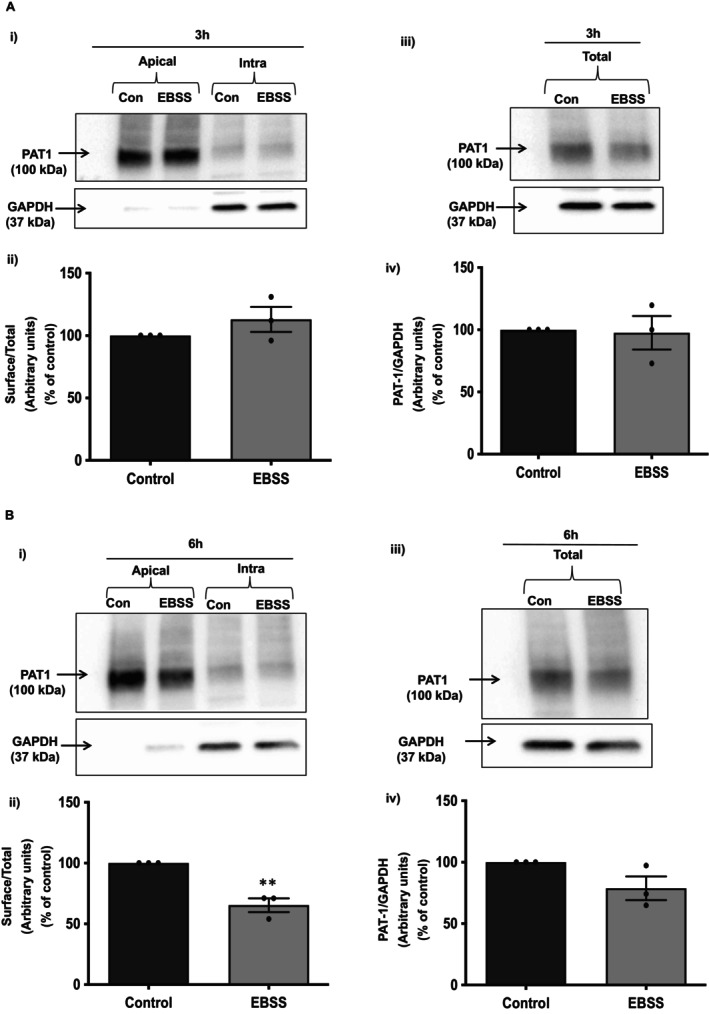
Nutrient starvation‐induced autophagy reduced apical surface expression of PAT‐1. Caco‐2 monolayers were nutrient starved for indicated time periods and apical membrane levels of PAT‐1 were determined by cell surface biotinylation. Surface and total levels of PAT‐1 probed by western blot, band intensities (*top panel*) and densitometric analysis (*bottom panel*) at 3 h (Ai–iv) and at 6 h (Bi–iv). GAPDH was used as internal control. *N* = 3, representative blots and images are shown. Results are mean ± SEM analysed by one‐way ANOVA. **p < 0.01 compared to control.

### 3.3 ATG7 Silencing Enhances PAT‐1 Protein Expression

Autophagy‐related genes (ATG) play an important role in the initiation and execution process of the autophagy response. In this regard, ATG7 is a core autophagy protein that plays a crucial role in LC3 (ATG8) lipidation and autophagosome formation [44, 45]. Loss of ATG7 impedes the autophagic pathway, resulting in an autophagy‐deficient state. To further test the role of autophagy in modulating PAT‐1 expression, we utilised predesigned siRNA to silence the ATG7 gene in Caco‐2 cells. As shown in Figure 3Ai, siRNA transfection resulted in a marked reduction in ATG7 mRNA (74%, *p* < 0.0001) normalised to GAPDH as an internal control compared with scrambled siRNA controls. PAT‐1 mRNA expression remained unaltered in the ATG7 siRNA transfected cells (Figure 3Aii). siRNA‐mediated knockdown of ATG7 expression was also confirmed at the protein level. Compared to the scrambled siRNA controls, ATG7 siRNA transfection resulted in an efficient knockdown of ATG7 protein levels (Figure 3Bi). Densitometric analysis showed ~70% reduction in ATG7 protein levels (Figure 3Bii) after silencing of ATG7 in Caco‐2 cells. Subsequently, the effect of ATG7 silencing was examined on LC3 and p62 protein levels. As shown in Figure 3Biii, ATG7 knockdown inhibited autophagy, as is evident from the impaired conversion of LC3 I to LC3 II (Figure 3Biv) and accumulation of p62 protein levels (Figure 3Bv) compared to cells transfected with scrambled siRNA. Silencing of ATG7 significantly increased PAT‐1 protein levels by ~50% (*p* < 0.01) (Figure 3Ci,ii) further confirming the novel role of autophagy in the post‐translational modulation of PAT‐1 expression.

### 3.4 Autophagy Reduces the Apical Membrane Protein Levels of PAT‐1

Autophagy has been shown to regulate the trafficking of the membrane proteins to the plasma membrane [29]. Previous studies have shown that PAT‐1 surface levels are regulated in response to various stimuli [15, 16]. Therefore, cell‐surface biotinylation studies were performed to examine the effect of autophagy induction on apical membrane levels of PAT‐1 protein. Caco‐2 monolayers were treated with EBSS for 3 and 6 h time points, followed by labelling with sulfo‐NHS‐SS‐Biotin. Our results showed that stimulation of autophagy via EBSS treatment significantly decreased the surface levels of PAT‐1 at 6 h (Figure 4Bi). Densitometric analysis of the protein bands suggested that EBSS treatment decreased surface PAT‐1 levels by ~40% compared to controls (Figure 4Bii). The total levels of PAT‐1 after 6 h of EBSS treatment showed a trend towards a decrease but did not reach significance (Figure 4Biii,iv). However, the total cellular and surface PAT‐1 levels did not change at 3 h (Figure 4Ai–iv). These results suggest that short‐term induction of autophagy in Caco‐2 cells with EBSS also decreased surface PAT‐1 levels.

### 3.5 In Vivo Effects of Autophagy Inhibition on PAT‐1 Expression

**FIGURE 5:**
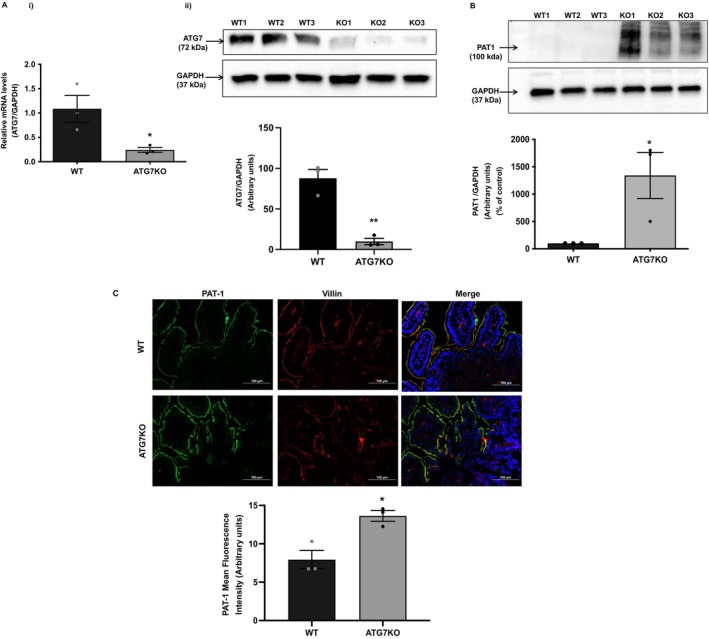
Genetic deficiency of autophagy enhances PAT‐1 levels in vivo. Genetic knockout of autophagy related protein, ATG7 (in ATG7KO mice compared to wildtype, WT mice) exhibits an expected reduction in ATG7 mRNA (Ai) and protein levels (Aii) (band intensities, Aii *upper panel*; and densitometric analysis, Aii *lower panel*). ATG7 knockout results in an increase in constitutive levels of PAT‐1 in the ileal region of ATG7KO mice compared to WT mice as determined by western blotting (band intensities, B *upper panel*; densitometric analysis, B *lower panel*) and immunofluorescence in ileal sections (representative image C *upper panel*; quantitative analysis C *lower panel*). GAPDH was used as an internal control. *n* = 3, representative blots and images are shown. Results are mean ± SEM analysed by one‐way ANOVA. *p < 0.05 and **p < 0.01 compared to wild type.

To assess the impact of autophagy in the native intestinal tissue on PAT‐1 expression, we utilised the transgenic murine model with ATG7 deficiency. Age‐matched ATG7 floxed [Atg7^fl/fl^, referred to as Wild type (WT)] and Atg7^−/−^ [referred to as ATG7KO (ATG7 knockout)] mice were used in the study. As PAT‐1 is predominantly expressed in the mouse small intestine, tissue lysates and OCT sections from the ileal region were used to determine PAT‐1 protein levels by western blotting and immunofluorescence, respectively. To validate the model, we first determined the loss of ATG7 mRNA and protein expression in WT and ATG7KO mice. A significant decrease in ATG7 mRNA levels (Figure 5Ai) and protein expression (Figure 5Aii) was noted in the ileum of ATG7KO mice compared to WT mice. Of note, ATG7 deficiency significantly increased the PAT‐1 protein levels (Figure 5B, *p* < 0.05) in ATG7KO mice compared to the WT mice. Effects of inhibition of autophagy (ATG7 deficiency) on PAT‐1 protein expression were further examined by immunofluorescence staining of ileal sections. Parallel to the western blotting data, suppression of autophagy significantly increased PAT‐1 levels (Figure 5C shown in green in the upper panel and quantitative analysis in the lower panel, *p* < 0.05) on the apical plasma membrane as shown by markedly increased co‐localisation (yellow) of PAT‐1 with villin (shown in red) in comparison to WT mice.

## 4 Discussion

Our current study demonstrates a novel mechanism of regulation of PAT‐1 expression in IECs by autophagy. Our data showed that induction of autophagy by nutrient deprivation or exposure to rapamycin decreased PAT‐1 protein levels in Caco‐2 cells. Conversely, inhibition of the autophagic pathway by ATG7 silencing resulted in increased PAT‐1 levels both in vitro in Caco‐2 cells and in vivo in ATG7KO mice.

Autophagy is a major cellular catabolic process essential for intestinal homeostasis [46], directing cytoplasmic contents, dysfunctional organelles, and pathogens to lysosomal degradation. At baseline, autophagy contributes to routine cell turnover and cellular homeostasis [47, 48]. In the intestine, autophagy is active in healthy mucosa and is essential for cell survival under stress [49]. Interestingly, autophagy has been shown to regulate the membrane expression and function of intestinal ion transporters and barrier proteins. For example, the transmembrane tight junction proteins, claudin‐2 and occludin, are reciprocally regulated by autophagy. Induction of autophagy enhances barrier function by increasing the lysosomal degradation of claudin‐2 (the pore‐forming claudin) while simultaneously preventing constitutive degradation of occludin [26, 33, 50]. Using nutrient starvation as a model to stimulate autophagy, we found that autophagic induction in Caco‐2 cells resulted in a significant decrease in PAT‐1 protein levels. However, this decrease in PAT‐1 levels was not limited to nutrient deprivation, as pharmacologic activation of autophagy by rapamycin mimicked a similar reduction in PAT‐1 protein expression. Conversely, genetic inhibition of autophagy by ATG7 siRNA increased PAT‐1 expression levels. It is interesting to note that in the renal tissue, calcium‐oxalate crystal‐mediated production of reactive oxygen species (ROS) results in autophagic activation and exacerbation of renal tubular epithelial cell injury [51]. Whether a similar phenomenon occurs in the gut under conditions of enteric hyperoxaluria remains unknown. However, conceiving similar oxidative stress due to (a) enteric hyperoxaluria and (b) persistent systemic and local inflammation, under conditions such as IBD and obesity [11], impairment in autophagy is expected. For example, in obesity, there is an increase in the level of pro‐inflammatory cytokines in the gut [52]. Proinflammatory factors have been shown to induce autophagy [53]. Thus, an already compromised PAT‐1 function and/or expression in a hyperoxaluric gut could be further augmented by induction of autophagy.

It is important to note that net oxalate transport involves the absorption of dietary oxalate and the secretion of non‐metabolised oxalate across the intestinal luminal membrane. This process is segment‐specific and occurs via transcellular and paracellular pathways, leading to either net absorption or secretion [14]. The key intestinal transporters involved in oxalate homeostasis include PAT1 (in the excretion of oxalate) and DRA (SLC26A3, in the absorption of oxalate) [54, 55].

In addition to PAT1 and DRA, other members of the SLC26 family, such as SLC26A1 (SAT1) and SLC26A2 (DTDST) also have the capacity to transport oxalate [13, 56]. However, SLC26A1 (localised at the basolateral membrane of the ileum and colon) does not appear to play any significant role in oxalate homeostasis, as SLC26A1 null mice do not exhibit hyperoxaluria or hyperoxalemia [57, 58]. Also, SLC26A2 (expressed at the apical membrane of IECs with higher levels in the colon compared to the small intestine) [59, 60] does not appear to play a significant role in intestinal oxalate transport [61] as mutations in the SLC26A2 gene have not been linked to hyperoxaluria or nephrolithiasis in mice or patients [59]. However, the detailed role of SLC26A2 in intestinal and renal oxalate transport remains to be explored. Since our study did not include functional transport assays or assess the expression of additional oxalate transporters mentioned above, we cannot conclusively determine the extent to which the inhibitory effect of autophagy induction on PAT‐1 alone contributes to impaired oxalate homeostasis. Future studies involving in vitro and in vivo functional assays, along with an assessment of the expression of other oxalate transporters in response to induced autophagy, will be necessary to pinpoint their role in the impairment of oxalate homeostasis.

In agreement with our in vitro studies, our in vivo studies utilising ATG7KO mice also showed that impairment of autophagic machinery resulted in increased levels of both total and apical membrane levels of PAT‐1 in the ileum. However, it should be noted that in our study, complete knockout of ATG7 was not achieved (~88% reduction in protein levels of ATG7). This partial reduction may be attributed to variability in tamoxifen‐induced gene knockout efficiency. While this reduction in ATG7 protein expression is substantial and likely impairs autophagy significantly, it is possible that residual ATG7 activity could still contribute to some level of autophagy. Also, the ATG7KO mice data in the current study showed results consistent with our in vitro study with siRNA‐mediated knockdown of ATG7 in Caco‐2 cells. These results indicate that even incomplete inactivation of ATG7 is potentially sufficient to impair autophagy and modulate PAT‐1 expression. Given the limitation of this mouse model, future studies utilising alternative genetic models, such as intestine‐specific ATG7 knockout or complementary approaches, like CRISPR/Cas9, are warranted to study the precise role of ATG7 in regulating PAT‐1 expression by autophagy‐mediated pathways.

In addition, our results also showed that basal autophagy was essential for PAT‐1 protein turnover. Cell‐surface biotinylation studies clearly showed reduced surface expression of PAT‐1 protein in response to autophagy induction. This, in conjunction with the data from ATG7KO mice and ATG7 silenced Caco‐2 cells, showed that basal autophagy plays an important role in the maintenance of both the total levels as well as the apical membrane levels of PAT‐1.

It is important to note that ubiquitination and autophagy are interconnected cellular pathways that often work together to regulate protein degradation and maintain cellular protein levels [62, 63]. Our findings indicate that the induction of autophagy negatively affects PAT‐1 expression, however, the potential role of ubiquitin‐mediated degradation in this process remains unclear. Further studies are warranted to determine whether PAT‐1 protein level is primarily regulated through the autophagy‐lysosome pathway or if the ubiquitin‐proteasome system also plays a role. Future studies examining the ubiquitination status of PAT‐1 in autophagy‐deficient models, such as ATG7 knockout mice, will be crucial for elucidating the precise mechanisms regulating its degradation under basal conditions.

In summary, our data, for the first time, demonstrated a constitutive role of basal autophagy in regulating PAT‐1 protein expression in intestinal epithelial cells. These findings are significant, considering that both insufficient and excessive autophagy is deleterious [64] and there is increasing evidence of IBD and obesity‐associated hyperoxaluria where PAT‐1 expression is compromised [11, 12, 65, 66]. The importance of autophagic regulation of intestinal PAT‐1 expression under pathophysiologic conditions manifesting hyperoxaluria warrants further research and highlights the potential importance of targeting autophagy in conditions of hyperoxaluria and kidney stones.

## Author Contributions

**Shubha Priyamvada:** conceptualization (lead), data curation (lead), formal analysis (lead), methodology (lead), project administration (lead), validation (lead), writing – original draft (lead), writing – review and editing (lead). **Dulari Jayawardena:** data curation (supporting), methodology (supporting), visualization (supporting). **Arivarasu N. Anbazhagan:** data curation (supporting), formal analysis (supporting), methodology (supporting), validation (supporting), writing – review and editing (supporting). **Anoop Kumar:** funding acquisition (supporting), data curation (supporting), formal analysis (supporting), methodology (supporting). **Seema Saksena:** funding acquisition (supporting), writing – review and editing (supporting). **Ravinder K. Gill:** funding acquisition (supporting), visualization (supporting), writing – review and editing (supporting). **Alip Borthakur:** conceptualization (supporting), writing – review and editing (supporting). **Waddah A. Alrefai:** funding acquisition (supporting), writing – review and editing (supporting). **Pradeep K. Dudeja:** conceptualization (lead), funding acquisition (lead), resources (lead), supervision (lead), writing – review and editing (lead).

## Disclosure

The authors have nothing to report.

## Conflicts of Interest

The authors declare no conflicts of interest.

## Data Availability

Data, analytic methods and study materials will be made available to other researchers via email.
